# An Unusual Case of Carbon Monoxide Poisoning from Formic and Sulfuric Acid Mixture

**DOI:** 10.5811/cpcem.2019.10.44265

**Published:** 2019-12-17

**Authors:** Muhammed Ershad, Athanasios Melisiotis, Zachary Gaskill, Matthew Kelly, Richard Hamilton

**Affiliations:** * Drexel University College of Medicine, Department of Emergency Medicine, Division of Medical Toxicology, Philadelphia, Pennsylvania; † Drexel University College of Medicine, Department of Emergency Medicine, Philadelphia, Pennsylvania; ‡ Penn Medicine, Division of Hyperbaric Medicine, Philadelphia, Pennsylvania

## Abstract

Formic acid, when combined with sulfuric acid, gets dehydrated to form carbon monoxide (CO). A 27-year-old female was found unconscious inside a car, next to a container with a mixture of sulfuric acid and formic acid. Concentrations of up to 400 parts per million of CO were measured inside the car post ventilation. Serum carboxyhemoglobin level was 15% after receiving 100% oxygen for two hours. The patient received hyperbaric oxygen therapy after which she was extubated with normal mental status. On follow-up after three months, she demonstrated neurocognitive abnormalities suggestive of delayed neurological sequelae from CO exposure.

## INTRODUCTION

Carbon monoxide (CO) toxicity is usually seen following exposure to smoke from house fires, heating system emissions, and exhaust fumes from motor vehicles. There are, however, various reported instances where CO produced through specific chemical reactions has been used by individuals to end their own lives. One such method involves combining formic acid with sulfuric acid, which produces CO.^1^ A total of 11 such reported cases were found in the literature, one of which was an outdoor occupational exposure.^2^–^10^ All the other cases were acts of suicide in enclosed surroundings including home spaces and, in one particular case, inside a car. We report a patient who generated CO from a sulfuric and formic acid mixture in her car in an attempt to commit suicide.

## CASE REPORT

A 27-year-old female was found unconscious in the front seat of her car. On the car floor was a five-gallon (18.9 liter [L]) plastic drum containing a funnel and hose. The drum contained a green oily fluid assumed to be a mixture of sulfuric acid and formic acid because empty containers of the same were found in the vicinity of the scene. First responders measured CO levels of 400 parts per million (ppm) inside the car, which was measured after adequate ventilation. On-scene assessment documented that the patient was minimally responsive to pain with occasional tonic-clonic movements of her extremities. Initial vitals were a blood pressure of 192/125 millimeters mercury, heart rate of 135 beats per minute, respiratory rate of 24 breaths per minute and a Glasgow Coma Scale of 8/15 (best eye response 2, best verbal response 2, best motor response 4). She was started on 100% oxygen via a non-rebreather mask and transferred to the emergency department (ED).

In the ED, she was noted to be minimally responsive with tonic clonic movements of her distal extremities. This was followed by decerebrate rigidity. Her pupils were symmetric and reactive to light bilaterally. The remainder of her neurological exam was unremarkable. She remained tachycardic. The patient was eventually intubated for airway protection because of her depressed mental status. Her initial labs revealed an elevated carboxyhemoglobin (COHB) level of 15% (0–3%), lactate of 2 millimoles (mmol)/L (0.5–2.2 mmol/L) and a troponin level of 3.066 nanograms per milliliters (ng/ml) (0–0.03 ng/ml). The electrocardiogram was normal.

The patient was transferred to a hyperbaric center around four hours after being found on the scene and almost immediately underwent three sessions of hyperbaric oxygen therapy over 24 hours. The first session was at 2.8 ATA (atmospheres absolute) for 45 minutes, 2.0 ATA for 60 minutes, and a five-minute air break. The second two cycles were at 2.0 ATA for 90 minutes. She was extubated the next day with normal mentation and neurological exam. Around four weeks after discharge, her neurologic evaluation demonstrated an anterograde amnesia beginning with the suicide event. In addition, she demonstrated other neurocognitive abnormalities suggestive of delayed neurological sequelae (DNS). Brain magnetic resonance imaging (MRI) showed abnormal restricted diffusion with associated fluid-attenuated inversion recovery (FLAIR) signal abnormalities in the white matter of the right temporal lobe, bilateral globus pallidi, bilateral mesial temporal lobes, hippocampus and scattered foci within the bilateral cerebellar hemispheres suggestive of anoxic-ischemic brain injury.

## DISCUSSION

Formic acid, when combined with sulfuric acid, gets dehydrated to produce CO. This method is used in the commercial production of CO in laboratories. In the past few years, this method has been used to commit suicide. One possible explanation could be the availability of a considerable number of books, webpages, and online forums that provide information on the production of CO for the purpose of suicide.^11^–^16^ Interestingly, another chemical reaction that has been recommended is heating calcium carbonate and zinc to produce calcium oxide, zinc oxide, and CO.^16^ It is important that first responders and emergency physicians be aware of such chemical reactions so that they can deploy appropriate on-scene and personal protection precautions.

Because CO is a colorless and odorless gas, it can be hazardous to first responders on the scene. A previous case report describes how a first responder developed CO poisoning in similar circumstances requiring several days of intensive critical care to recover.^9^ In most of the reported cases, the victims had displayed warning signs outside their enclosures, warning the first responders against CO exposure. In our case, the first responders were initially unaware of a potential hazardous materials (HAZMAT) situation on the scene and hence were not adequately protected with personal protective equipment. They reported a “chemical smell” when they initially got into the car. There were, however, no reported injuries among them on later assessment. The HAZMAT team was later deployed on the scene, and after ventilating the car they identified the chemicals and measured the CO levels. Considering the CO levels were measured after appropriate ventilation of the car, the levels measured (400 ppm) were most likely an underestimation of the maximum CO concentration.

The patient was not decontaminated on the scene due to the initial lack of information about the HAZMAT involved. The ED was alerted that the patient originated in an unknown HAZMAT scene prior to patient arrival and hence the patient was decontaminated with tepid water and soap in a stand-alone decontamination room prior to entering the ED.

**CPC-EM Capsule**

What do we already know about this clinical entity?

Formic acid when combined with sulfuric acid generates carbon monoxide (CO) as a byproduct which has been reported as a method of suicide in various case reports.

What makes this presentation of disease reportable?

This is the first case reported where hyperbaric oxygen (HBO) therapy was used to treat such a unique form of CO poisoning. In spite of timely initiation with HBO therapy, the patient still developed delayed neurological sequelae.

What is the major learning point?

Delayed neurological sequlae can occur despite timely intervention with HBO therapy. Combining formic acid with any strong acid can potentially generate dangerous concentrations of CO, especially in enclosed spaces.

How might this improve emergency medicine practice?

Emergency physicians can be made more aware of this chemical reaction, which may help in earlier identification of potential CO toxicity and appropriate allocation of triage resources especially in a mass casualty incident.

The initial serum COHB level measured in the ED was 15.1%, more than two hours after the patient was started on 100% oxygen. Considering that the half-life of COHB is around 75 minutes on 100% oxygen at atmospheric pressure,^17^ it is possible that the patient had a COHB level in the range of 45% when she was found in the car, assuming zero order kinetics.^18^ Use of a simplified version of the Coburn-Forster-Kane model predicts that the patient had a COHB level of 38.3 % [(COHB(%) = 100/[1 + R(643/ppm CO)].^19^ Previous studies have interestingly indicated poor correlation between initial COHB levels, clinical manifestations, and the risk of delayed neurological sequelae. This patient would serve as an example to this fact, considering that the initial COHB levels were only moderately elevated despite the remarkable clinical presentation and noteworthy delayed neurological sequelae. It is also not clear how long the patient was exposed to CO inside the car before being evacuated, because the patient herself had poor recall of the events.

After resolution of the acute clinical course, the patient was admitted to psychiatry where she underwent treatment for depression. Four weeks after the exposure, she developed retrograde amnesia. She had no awareness of the suicide event or the events leading up to the suicide and demonstrated euthymia, inappropriate laughter, incongruent affect, childlike behavior, and impaired short- and long-term memory. She endorsed to having been treated for drug-resistant depression with ketamine infusions in the past but could not recall suicidality or plans of suicide around the time of the suicide attempt. She reported having no feelings of being suicidal a month after the event. At three months follow-up, many of her cognitive deficits had improved but her amnesia remained.

Delayed neurological sequelae following CO poisoning can present with a multitude of neurological and cognitive symptoms and signs. DNS may develop in up to 40% of the survivors of acute CO poisoning within 2–40 days.^20^ A Cochrane systematic review analysis concluded that there is insufficient evidence to support the use of hyperbaric oxygen (HBO) to prevent DNS from CO poisoning.^21^ Similarly, the American College of Emergency Physicians has noted that there remains lack of clarity over whether HBO is superior to normobaric oxygen for improving long-term neurocognitive outcomes in CO exposure.^22^ Our patient developed DNS despite timely HBO therapy. This is the first case report of poisoning from chemical production of CO where long-term follow-up revealed delayed neurocognitive manifestations and distinct MRI findings in the face of expedient HBO therapy.

## CONCLUSION

The case presented here demonstrates an unusual way of attempting suicide by combining formic acid with sulfuric acid to generate CO inside an enclosed car. The patient was treated with hyperbaric oxygen and survived the event but developed delayed neurological sequelae. Emergency physicians and first responders need to be aware of such chemical reactions to avoid injury to first responders and to guide appropriate treatment.
